# Supplementary material for: nf-core/mag: a best-practice pipeline for metagenome hybrid assembly and binning
Source: NAR Genom Bioinform. 2022 Feb 2;4(1):lqac007. doi: 10.1093/nargab/lqac007 (PMC8808542; doi:10.1093/nargab/lqac007)
Supplement: lqac007_Supplemental_File [file lqac007_supplemental_file.pdf]

nf-core/mag: a best-practice pipeline for metagenome hybrid assembly and binning

Supplementary material

Sabrina Krakau[1,2,*], Daniel Straub[1,3], Hadrien Gourlé[4], Gisela Gabernet[1] and Sven Nahnsen[1,2,5]

[1]Quantitative Biology Center (QBiC), University of Tübingen, Tübingen, Germany, [2]Cluster of Excellence – Controlling Microbes to Fight Infections, University of Tübingen, Tübingen, Germany, [3]Microbial Ecology, Center for Applied Geosciences, University of Tübingen, Tübingen, Germany, [4]Department of Animal Breeding and Genetics, Swedish University of Agricultural Sciences, Uppsala, Sweden, [5]Biomedical Data Science, Department of Computer Science, University of Tübingen, Tübingen, Germany

* To whom correspondence should be addressed. Email: sabrina.krakau@qbic.uni-tuebingen.de

1. Comparison of existing pipelines for metagenome assembly and binning

Table S1 shows a comparison of nf-core/mag to existing pipelines that are implemented using workflow management systems, such as Snakemake or Nextflow, and allow a scalable and easy to use application on HPC clusters. Note that this comparison is focused on pipelines for the assembly and binning of metagenomes and does not include pipeline features for other analysis types.

Table S1: Comparison of metagenome assembly and binning pipelines.

	**Feature**	**metagWGS v2.0** (1)	**Muffin v1.0.3** (2)	**ATLAS v2.6a2** (3)	**nf-core/mag v2.1.0**
	Workflow management system	Nextflow	Nextflow	Snakemake	Nextflow
Read QC	Supported sequencing technologies	Illumina	Illumina, Nanopore	Illumina	Illumina, Nanopore
	Adapter and quality Trimming	Cutadapt, Sickle	fastp, Filtlong	BBTools suite	fastp, Porechop, Filtlong
	PCR duplicate removal	BWA + Bedtools	No	BBTools suite	No
	Host-read removal	BWA + Bedtools	No	BBTools suite	Bowtie2
Assembly	Short read assembly	metaSPAdes, MEGAHIT	metaSPAdes	metaSPAdes, MEGAHIT	metaSPAdes, MEGAHIT
	Long read assembly	No	MetaFlye	No	No
	Hybrid assembly	No	hybridSPAdes	hybridSPAdes (but no preprocessing for long reads)	hybridSPAdes

	Assembly refinement	Filters contigs based on mapped reads	MetaFlye assembly: Racon, Medeka, Pilon<br>Reassembly after binning: Unicycler	Filters contigs based on mapped reads and contig lengths	No
	QC	metaQUAST	No	No	metaQUAST
	Group-wise co-assembly	No	No	No	Yes
Genome binning	Binning	MetaBAT2	MetaBAT2, MaxBin2, CONCOCT	MetaBAT2, MaxBin2	MetaBAT2
	Group-wise co-abundances used for binning	No	No	Yes	Yes
	QC	BUSCO, metaQUAST (copied from nf-core/mag)	CheckM	CheckM	BUSCO, metaQUAST
	Bin refinement	No	MetaWRAP	DAS Tool	No
	Dereplication	No	No	dRep	No
	MAG abundance estimation	Yes	No	Yes	Yes
Annotation	Gene prediction	Prokka	No	Prodigal	Prodigal (run by BUSCO)
	Taxonomic classification	CAT/BAT	Sourmash with GTDB	GTDB-Tk	GTDB-Tk, CAT/BAT
	Functional annotation	eggNOG	eggNOG	eggNOG	No
Usability	Documentation	Yes	Yes	Yes (dedicated documentation of parameters missing)	Yes
	Aggregated QC results	MultiQC	No	No	MultiQC
	Interactive user support	GitLab Issues	GitHub Issues	GitHub Issues	GitHub Issues, Slack channel
	Reproducibility	No (no option to run MetaBAT2 in deterministic setting)	No (no option to run MetaBAT2 in deterministic setting)	No (no option to run MetaBAT2 in deterministic setting)	Yes
	Continuous integration tests	No	No	CircleCI	Small tests on GitHub Actions, “full-size” tests on AWS
	Launching and monitoring of pipeline runs via web interface	No	No	No	Nextflow tower (on local computers, cluster or cloud systems)

In the following we briefly discuss the tool choices for nf-core/mag v2.1.0 with respect to its core functionalities.

Assembly. To compute assemblies based on short reads, the state-of-the-art assembly tools metaSPAdes and MEGAHIT can be used within nf-core/mag. Both tools show a consistent high performance across a variety of datasets in the CAMI II challenge. MEGAHIT has very efficient memory (RAM) management, while metaSPAdes has a higher memory requirement, which can cause problems particularly for the computation of large metagenomes or co-assemblies. To compute hybrid assemblies based on short and long reads, hybridSPAdes is used. In case within the community there will be the need for assemblies based solely on long reads (for example when Nanopore sequencing becomes cheaper, allowing higher sequencing depths), the pipeline can be extended with tools such as metaFlye (4).

Binning. To reconstruct MAGs, the pipeline was implemented to use the binning tool MetaBAT2 based on user requests. In the CAMI II challenge, it was shown that MetaBAT2 has a higher purity compared to other binning tools such as MaxBin2 (5) or CONCOCT (6), while reaching a lower completeness than CONCOCT and a lower ARI (adjusted Rand index) than MaxBin2. In the future, the pipeline can be extended to include different binning tools that can be used depending on the experimental requirements, or to additionally use bin refinement tools such as DAS Tool (7) (which combines the results from multiple binning methods to further improve the quality).

The quality of the retrieved bins is assessed with BUSCO v5. Another widely used tool to assess the bin quality is CheckM (8), which makes use of lineage-specific marker genes. Both tools seem to perform comparably (9). While CheckM can handle bacterial and archaeal genomes, BUSCO v5 can additionally assess the quality of eukaryotic genomes as well as of genomes for a subset of viruses. Furthermore, since version 5, BUSCO can automatically select for each bin the most specific lineage dataset (containing the single-copy orthologs for benchmarking), aiming to increase the resolution and allowing the analysis for genomes of unknown origin (9).

Taxonomic classification. MAGs can be taxonomically classified in this pipeline with CAT/BAT or GTDB-TK. For this, CAT/BAT aligns predicted protein sequences against the NCBI non-redundant reference database (10), while GTDB-TK uses a set of marker genes. Thus, GTDB-TK is computationally more efficient than CAT/BAT, but can only classify MAGs that pass a certain quality threshold. Another important aspect is that GTDB-TK allows easy maintenance and reproducibility, since it is bi-annually updated together with the GTDB (11) and older database versions remain accessible. Besides CAT/BAT and GTDB-TK, the tool PhyloPhlAn 3.0 (12) was developed for taxonomic classification and published around a similar time, and it remains to be evaluated how these tools perform in comparison.

Additional pipeline extensions for functional annotation as well as for assembly and bin refinement steps, as partly already implemented in Muffin and ATLAS, are envisioned for future releases.

2. nf-core and DSL2

All nf-core pipelines must be based on the nf-core template. This template was recently ported to the new Nextflow DSL2 syntax, which enables a modularised structure and reuse of components, with each process using its own BioContainer (13). nf-core/mag is ported to DSL2 since version 2.0.0. For a detailed description of the nf-core framework see the main nf-core publication (14) or the nf-core website (https://nf-co.re).

3. Reproducibility

Generating results that can be reproduced is a major challenge and many findings published in scientific literature can still not be replicated by other scientists (15). The nf-core framework enables reproducibility as described in the 'Material and Methods' section. To additionally ensure that the individual tools generate reproducible results, several reproducibility settings were implemented for nf-core/mag. MEGAHIT and SPAdes, for example, depend on multi-threading parameters and the number of CPUs used for computation can affect the final results. In nf-core/mag, the number of used CPUs can be fixed and reported accordingly to generate reproducible assemblies. This ensures that the specified number of CPUs is not increased in case these processes will be re-submitted (as is usually the case for nf-core pipelines, if the specified resource requirements for a process do not suffice). For MetaBAT2, a deterministic behaviour is enabled by default within this pipeline via a fixed seed parameter. Moreover, specific settings allow the generation and/or saving of databases for BUSCO or CAT, for which the required public databases do not always remain accessible.

4. Simulated metagenomic data

The metagenomic data was simulated with the most recent development version of CAMISIM at the time of preparing this article (available at https://doi.org/10.5281/zenodo.5137751)(16). Two groups of samples were generated by using different CAMISIM seeds ('seed=1000' and 'seed=1001'), each comprising a time series of four samples. To simulate Illumina reads, the parameters 'ncbi_taxdump=tools/ncbi-taxonomy_20180226.tar.gz', 'number_of_samples=4', 'genomes_total=500', 'genomes_real=500', 'mode=timeseries_lognormal', 'gauss_mu=1' and 'gauss_sigma=1' were specified in addition to the seed and the default parameters in the CAMISIM configuration file 'defaults/default_config.ini'. The simulated FASTQ datasets were split with respect to paired-end reads. For the simulation of Nanopore reads, CAMISIM was run in combination with NanoSim v2.5.0. The parameters 'anonymous=False', 'ncbi_taxdump=tools/ncbi-taxonomy_20180226.tar.gz', 'number_of_samples=4', 'genomes_total=500', 'genomes_real=500', 'mode=timeseries_lognormal', 'gauss_mu=1' and 'gauss_sigma=1' were specified in addition to the seed and the default parameters in the configuration file 'defaults/nanosim_config.ini'. For each sample the resulting genome-wise FASTQ files were merged.

5. Results on simulated data

We ran nf-core/mag with the different assembly settings on the simulated metagenomic data. The following command was used (Nextflow v21.04.1) to generate short read and hybrid sample-wise assemblies:

```
> nextflow run nf-core/mag -r 2.1.0 -profile cfc -c custom.config --input
samplesheet.CAMISIM_hybrid.csv --binning_map_mode all --spades_fix_cpus 40
--spadeshybrid_fix_cpus 40 --skip_megahit
```

Corresponding group-wise co-assemblies were generated with:

```
> nextflow run nf-core/mag -r 2.1.0 -profile cfc -c custom.config --input
samplesheet.CAMISIM_hybrid.csv --coassemble_group --binning_map_mode all
--spades_fix_cpus 40 --spadeshybrid_fix_cpus 40 --skip_megahit
```

A ‘custom.config’ file was used to increase the memory for SPAdes and contained:

```
process {
  withName: SPADES {
    memory        = 150.GB
  }
  withName: SPADESHYBRID {
    memory        = 150.GB
  }
}
```

Besides comparing the resulting assemblies (see Figure 2), we compared the reconstructed genomes with respect to commonly used MAG metrics. The results shown in Figure S1 demonstrate that the average number of contigs per MAG decreased with hybrid vs. short read assembly. However, the average quality across all MAGs does not increase when using the hybrid or co-assembly assembly setting. For example, performing group-wise co-assemblies results in a higher average contamination compared to sample-wise assemblies (see Figure S2 d)), and hybrid assemblies result in lower completenesses compared to short read assemblies (see Figure S2 c)). This is likely caused by the highly increased number of reconstructed MAGs when using these settings (see Figure 2E).

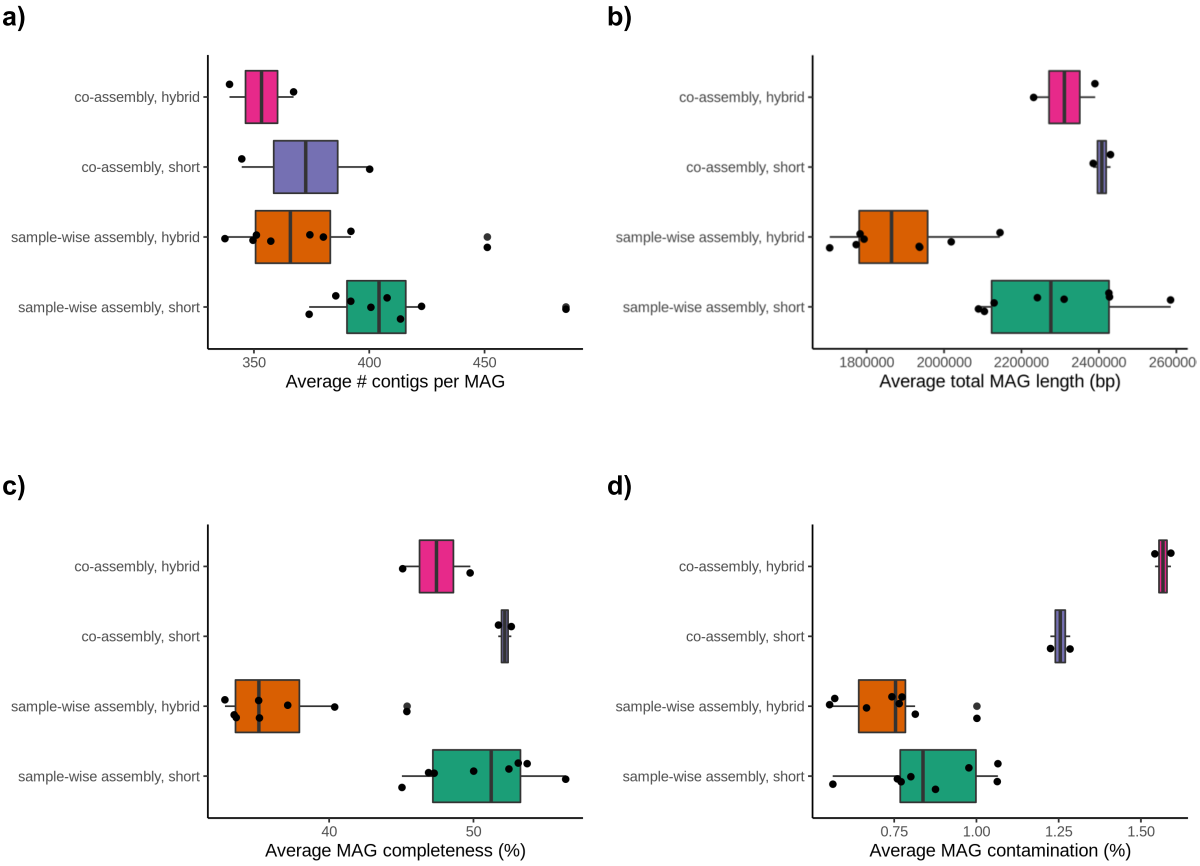

Figure S1: MAG-wise metrics obtained using different nf-core/mag assembly settings on the simulated data: sample-wise assembly, group-wise co-assembly, short read only assembly or hybrid assembly. Each point corresponds to one assembly, originating either from one sample or one group. Metrics displayed are **a)** average number of contigs per MAG, **b)** average total MAG length in base pairs, **c)** average MAG completeness and **d)** average MAG contamination. The number of contigs and the total length of each MAG were summarised by QUAST, the MAG completeness and contamination were estimated by BUSCO.

References

1. Fourquet, J., Noirot, C., Klopp, C. C., Pinton, P., Combes, S., Hoede, C. and Pascal, G. (2020). Whole metagenome analysis with metagWGS [Poster]. *JOBIM2020*.
2. Van Damme, R., Hölzer, M., Viehweger, A., Müller, B., Bongcam-Rudloff, E. and Brandt, C. (2021). Metagenomics workflow for hybrid assembly, differential coverage binning, metatranscriptomics and pathway analysis (MUFFIN). *PLOS Computational Biology*, 17(2), e1008716.
3. Kieser, S., Brown, J., Zdobnov, E. M., Trajkovski, M. and McCue, L. A. (2020). ATLAS: A Snakemake workflow for assembly, annotation, and genomic binning of metagenome sequence data. *BMC Bioinformatics*, 21, 257.

4. Kolmogorov, M., Bickhart, D. M., Behsaz, B., Gurevich, A., Rayko, M., Shin, S. B., Kuhn, K., Yuan, J., Polevikov, E., Smith, T. P.L. et al. (2020). metaFlye: scalable long-read metagenome assembly using repeat graphs. *Nature Methods*, 17(11), 1103-1110.
5. Wu, Y. W., Simmons, B. A. and Singer, S. W. (2016). MaxBin 2.0: an automated binning algorithm to recover genomes from multiple metagenomic datasets. *Bioinformatics*, 32(4), 605-607.
6. Alneberg, J., Bjarnason, B. S., De Bruijn, I., Schirmer, M., Quick, J., Ijaz, U. Z., Lahti, L., Loman, N. J., Andersson, A. F. and Quince, C. (2014). Binning metagenomic contigs by coverage and composition. *Nature Methods*, 11(11), 1144-1146.
7. Sieber, C. M., Probst, A. J., Sharrar, A., Thomas, B. C., Hess, M., Tringe, S. G. and Banfield, J. F. (2018). Recovery of genomes from metagenomes via a dereplication, aggregation and scoring strategy. *Nature Microbiology*, 3(7), 836-843.
8. Parks, D. H., Imelfort, M., Skennerton, C. T., Hugenholtz, P. and Tyson, G. W. (2015). CheckM: assessing the quality of microbial genomes recovered from isolates, single cells, and metagenomes. *Genome Research*, 25(7), 1043-1055.
9. Manni, M., Berkeley, M. R., Seppey, M., Simao, F. A. and Zdobnov, E. M. (2021). BUSCO Update: Novel and Streamlined Workflows along with Broader and Deeper Phylogenetic Coverage for Scoring of Eukaryotic, Prokaryotic, and Viral Genomes. *Molecular Biology and Evolution*, 38(10), 4647–4654.
10. Sayers, E. W., Beck, J., Bolton, E. E., Bourexis, D., Brister, J. R., Canese, K., Comeau, D. C., Funk, K., Kim, S., Klimke, W. et al. (2021). Database resources of the national center for biotechnology information. *Nucleic Acids Research*, 49(D1), D10.
11. Parks, D. H., Chuvochina, M., Rinke, C., Mussig, A. J., Chaumeil, P. A. and Hugenholtz, P. (2021). GTDB: an ongoing census of bacterial and archaeal diversity through a phylogenetically consistent, rank normalized and complete genome-based taxonomy. *Nucleic Acids Research*, 50(D1), D785-D794.
12. Asnicar, F., Thomas, A. M., Beghini, F., Mengoni, C., Manara, S., Manghi, P., Zhu, Q., Bolzan, M., Cumbo, F., May, U. et al. (2020). Precise phylogenetic analysis of microbial isolates and genomes from metagenomes using PhyloPhlAn 3.0. *Nature Communications*, 11, 2500.
13. da Veiga Leprevost, F., Grüning, B. A., Alves Aflitos, S., Röst, H. L., Uszkoreit, J., Barsnes, H., Vaudel, M., Moreno, P., Gatto, L., Weber, J., Bai, M., Jimenez, R. C., Sachsenberg, T., Pfeuffer, J., Vera Alvarez, R., Griss, J., Nesvizhskii, A. I. and Perez-Riverol, Y. (2017). BioContainers: An open-source and community-driven framework for software standardization. *Bioinformatics*, 33(16), 2580–2582.
14. Ewels, P. A., Peltzer, A., Fillinger, S., Patel, H., Alneberg, J., Wilm, A., Garcia, M. U., Di Tommaso, P. and Nahnsen, S. (2020). The nf-core framework for community-curated bioinformatics pipelines. *Nature Biotechnology*, 38(3), 276–278.
15. Baker, M. (2016). 1,500 scientists lift the lid on reproducibility. *Nature*, 533(7604), 452–454.
16. Fritz, A., Hofmann, P., Belmann, P., Bremges, A., McHardy, A., Dröge, J. and DeMaere, M. (2021). skrakau/CAMISIM: Simulation of hybrid, time series data. *Zenodo*.
